# Risk Factors of Developmental Defects of Enamel-A Prospective Cohort Study

**DOI:** 10.1371/journal.pone.0109351

**Published:** 2014-10-02

**Authors:** Hai Ming Wong, Si-Min Peng, Yi Feng Wen, Nigel M. King, Colman P. J. McGrath

**Affiliations:** 1 Paediatric Dentistry & Orthodontics, Faculty of Dentistry, The University of Hong Kong, Hong Kong; 2 Paediatric Dentistry, School of Dentistry, University of Western Australia, Perth, Australia; 3 Dental Public Health, Faculty of Dentistry, The University of Hong Kong, Hong Kong; 4 Department of Paediatric Dentistry, Guanghua School of Stomatology, Hospital of Stomatology, Sun Yat-sen University, Guangzhou, China; University of North Carolina at Chapel Hill, United States of America

## Abstract

**Background and objective:**

Current studies on the aetiology of developmental defects of enamel (DDE) are subject to recall bias because of the retrospective collection of information. Our objective was to investigate potential risk factors associated with the occurrence of DDE through a prospective cohort study.

**Methods:**

Using a random community sample of Hong Kong children born in 1997, we performed a cohort study in which the subjects’ background information, medical and dental records were prospectively collected. A clinical examination to identify DDE was conducted in 2010 when the subjects were 12 years old. The central incisor, lateral incisor and first molar in each quadrant were chosen as the index teeth and were examined ‘wet’ by two trained and calibrated examiners using the modified FDI (DDE) Index.

**Results:**

With a response rate of 74.9%, the 514 examined subjects had matched data for background information. Diffuse opacites were the most common type of DDE. Of the various possible aetiological factors considered, only experience of severe diseases during the period 0–3 years was associated with the occurrence of ‘any defect’ (p = 0.017) and diffuse opacities (p = 0.044). The children with experience of severe diseases before 3 years of age were 7.89 times more likely to be affected by ‘any defect’ compared with those who did not have the experience (OR 7.89; 95% CI 1.07, 58.14; p = 0.043). However, after adjusting for confounding factors, the association no longer existed.

**Conclusion:**

No variables could be identified as risk factors of DDE in this Hong Kong birth cohort.

## Introduction

Developmental defects of enamel (DDE) are regions of enamel with altered quality and quantity as a consequence of insults to the enamel organ at the time of enamel formation [1]. DDE occur frequently on the incisors [2], [3], and may thereby lead to major aesthetic concerns [4]. Common complications of DDE include tooth sensitivity and occlusal dysfunction [4], [5]. Furthermore, DDE have been reported to predispose the teeth to early childhood caries [6] and increased attrition [7]. Despite the distinct treatment need of teeth affected by DDE, it is unfortunate that the treatment has frequently been unsuccessful because the altered enamel structure in DDE creates difficulties with anaesthetizing the tooth and bonding of the restoration to the enamel [5].

Faced with such a dilemma, we may consider directing our focus to the prevention of DDE, which in itself requires a substantial understanding of relevant aetiological factors. Although the aetiology of DDE has been studied for many years and that over 90 different factors have been identified [8], [9], the best available data have been gained from animal studies and individual case reports of children with systemic disorders. Thus, reliable evidence for the involvement of specific risk factors remains equivocal.

According to published studies seeking possible risk factors for DDE, low socio-economic status, respiratory infections, exposure to cigarette smoking, asthma, otitis media, urinary tract infection, chickenpox and early life health problems have been said to be associated with enamel defects [10]–[14]. However, these results have always been challenged. For example, while amoxicillin has been reported to significantly increase the probability of DDE [15], [16], such an effect was not found among a random sample of Western Australian children [17]. Similar conflicting results have also been reported for the impacts of otitis media [10], [12], chicken pox [12], [17], and maternal factors [16], [17]. Furthermore, some New Zealand researchers failed correlate any early life health problems and the occurrence of DDE in the permanent dentition [18].

Conclusions from these studies should therefore be interpreted with caution. Primarily because information about past childhood diseases was mainly collected retrospectively after a relatively long interval and was hence dependent on the reliability of parental recall which can be notoriously poor. In addition, small sample sizes and the low prevalence of DDE limit the ability to differentiate the effects of the exposures on the creation of specific type of defects. Hence, the lack of robust data makes the results of these studies inconclusive.

The paucity of reliable information on possible causal factors of DDE supports the need for further investigations into the causes of these defects. Thus, the present study aimed to investigate the prevalence of DDE among a random group of Hong Kong adolescents aged 12 years old and to explore potential risk factors associated with the occurrence of DDE in these children who had prospectively maintained health records.

## Methods

### Ethics Statement

The study protocol was approved by the Institutional Review Board of the University of Hong Kong/Hospital Authority Hong Kong West Cluster (IRB reference number: UW 09-437, UW 11-070).

### Patient Selection

This was a prospective, population-representative study. The eligible subjects were Hong Kong children born during April and May of 1997 when the fluoride in public water supply system was 0.5 ppm. The study was carried out in 2010 when the subjects were 12 years old. The sampling frame was all local secondary schools in Hong Kong (by law all children are required to attend secondary school). A random sample of 45 schools (approximately 10% of all local secondary schools) from 18 districts in Hong Kong, SAR, was selected. The secondary schools were the primary sampling unit. Within each school all Form 1 and Form 2 (equivalent to US Grade 6 and 7) students born between April 1st and May 31st, 1997 were invited to participate in the study. Parents/primary caregivers provided their written consent and students were asked to provide their assent. A sample size of 497 was calculated based on the prevalence of DDE at 89% in Hong Kong adolescents [19], and an odds ratio of 3.31 for having DDE among subjects with and without infection during the neonatal period [17]. The statistical power was 0.8 and the significance level was set at 0.05. Anticipating a response rate of 75%, 668 subjects were invited to participate in the study.

### Information Collection

Background information solicited from the parents/guardians covered several aspects including socio-economic status, family history, infant and early childhood exposures, and oral hygiene habits. In addition, medical and dental records of the children, in particular their birth characteristics and history of oral diseases were also sought. Health care background information and data were obtained for these children immediately after birth and then on yearly basis through active follow-up surveys in the form of self-reported questionnaires; medical and dental records of the subjects were retrieved by passive follow-up via record linkage [20].

### DDE Clinical Examination

Examinations were performed on the central incisors, lateral incisors and first permanent molars of the subjects. Two trained and calibrated examiners, who were blind to the subjects’ information and the objectives of this study, completed all of the clinical examinations. Approximately 5% of the children were re-examined to test the intra- and inter-examiner reliabilities.

The children were instructed to lie in a supine position on a portable dental chair in classrooms in the schools. Before the examination, all of the incisors and first molars were cleaned with gauze in order to remove any gross plaque or food deposits that may have been present. These teeth were then examined under ‘wet’ conditions using a plane intra-oral disposable mouth mirror with a built in LED light source and a blunt probe. The probe was used to detect, or confirm the presence of any discontinuity of the enamel surface of the teeth.

### Diagnostic Criteria of DDE

The diagnostic criteria were based on the modified version of the FDI (DDE) Index for use in general purpose epidemiological studies [21]. Three main types of enamel defects, based on their macroscopic appearance, were recognized, namely demarcated opacities, diffuse opacities and hypoplasia [21]. The demarcated opacities included the white/cream and the yellow/brown subtypes. Under the main type of diffuse opacities, there were subtypes of diffuse lines, diffuse patchy, diffuse confluent, and confluent/patchy plus staining and/or loss of enamel. Hypoplasia included subtypes of pits and missing enamel.

### Statistical Analysis

Sample size was calculated using SAS software for windows, v9.2 (SAS Institute, Inc., CARY, NC, USA). Data collected were coded and entered into IBM SPSS Statistics v20.0 (IBM Corp., Armonk, NY, USA) for analysis. Information on the DDE status at the tooth surface level was used to generate information at the tooth level and the subject level. In the analysis, the eight different subtypes of DDE recorded were grouped into the three main types. ‘Any defect’ denoted the presence of DDE regardless of the main types. Frequency distributions of DDE by type at tooth and subject level were calculated. Bivariate associations between the occurrence of DDE and potential causal factors were tested using chi-square test for categorical independent variables and ANOVA for continuous independent variables. The statistical significance level was set at 0.05.

To assess the relative strength of the association between the presence of different types of DDE and various potential risk factors, unadjusted and adjusted logistic regression analyses were performed. In the unadjusted model, separate logistic regression was undertaken for each individual aetiological factor; while for the adjusted model, all explanatory variables were simultaneously entered into the model and a backward stepwise logistic regression was performed. The model building strategy for the adjusted logistic regression analysis was as follows: (i) variables with a p value no greater than 0.25 in bivariate analysis were included in the regression model; and (ii) at the significance level of 0.05, variables that did not contribute to the model as calculated by the likelihood ratio test were backward eliminated, until all retained variables were of significant impact on the model. For both models, the unadjusted and adjusted odds ratio (OR) and 95% confidence intervals (CI) were estimated. Poisson regression models were also been conducted to assess the association between the rarer main types of DDE and potential causal factors.

## Results

There were a total of 668 children who had been randomly selected into this study and all of them completed the clinical examinations. Among these children, 514 had the matched background information, which represented a response rate of 76.9%. Twenty seven subjects (756 tooth surfaces) were re-examined to measure the level of examiner reliability throughout the study. At the tooth surface level, the intra-examiner reliabilities for the diagnoses of the various subtypes of DDE were 0.87 and 0.85, while the inter-examiner reliability was 0.82 (unweighted κ values).

Table 1 shows the frequency distribution of various types of DDE by types of tooth and at the subject level. Diffuse opacities were found to be the most prevalent of the three main types of DDE both at the tooth and at subject level. First permanent molars were more frequently affected by DDE than the incisors.

**Table 1:** Frequency distribution [n (%)] of DDE by type at tooth and subject level.

	Tooth level																			
	Central incisor						Lateral incisor						1^st^ permanent molar							
Type of DDE	Upper(N = 1000)		Lower(N = 988)		Total(N = 1988)		Upper(N = 1000)		Lower(N = 981)		Total(N = 1981)		Upper(N = 1002)		Lower(N = 1004)		Total(N = 2006)		Subject level(N = 514)	
No defects	200	(19.2)	430	(46.5)	630	(31.7)	219	(21.9)	380	(38.7)	599	(30.2)	157	(15.7)	241	(24.0)	398	(19.8)	52	(10.1)
Any defects	800	(80.8)	558	(56.5)	1358	(68.3)	781	(78.1)	601	(61.3)	1382	(69.8)	845	(84.3)	763	(76.0)	1608	(80.2)	462	(89.9)
Demarcatedopacities	20	(2.0)	17	(1.7)	37	(1.9)	2	(0.2)	3	(0.3)	5	(0.3)	11	(1.1)	16	(1.6)	27	(1.3)	44	(8.6)
Diffuseopacities	795	(79.5)	555	(56.2)	1350	(67.9)	780	(78.0)	600	(61.2)	1380	(69.7)	844	(84.2)	757	(75.4)	1601	(79.8)	460	(89.5)
Hypoplasia	0	(0)	4	(0.4)	4	(0.2)	0	(0)	1	(0.1)	1	(0.1)	5	(0.5)	2	(0.2)	7	(0.3)	9	(1.8)

Bivariate associations of the occurrence of DDE and each independent variable are presented in Table 2. A higher proportion of demarcated opacities was observed among male subjects (p<0.05), while no gender difference was found for ‘any defect’, diffuse opacities or hypoplasia (p>0.05). The experience of severe diseases during the 0–3 year period was associated with a higher prevalence of ‘any defect’ (p = 0.017) and diffuse opacities (p = 0.044). The reported severe diseases prior to 3 years of age included spinal bifida, congenital heart disease, neonatal lupus erythematosus, Kawasaki disease, thalassemia, hepatitis B, meningitis, pneumonia, nephritis, pancreatitis, and severe asthma. While no statistical significant associations were found for other variables, higher proportion of ‘any defect’ and diffuse opacities were observed among subjects with lower levels of household monthly income (p>0.05). In addition, children who used an adult type toothpaste before 5 years of age were more susceptible to ‘any defect’ and diffuse opacities than those who used a toothpaste formulated for children (p>0.05).

**Table 2:** Distribution of variables among children with different types of DDE.

Variable	No defects		Any defects		Demarcated opacities		Diffuse opacities		Hypoplasia	
Gender (N = 514)										
Male	28	(10.8)	231	(89.2)	30	(11.6)*	229	(88.4)	4	(1.5)
Female	24	(9.4)	231	(90.6)	14	(5.5)*	231	(90.6)	5	(2.0)
Gestation; weeks (N = 514)^a^	39.1	(1.6)	38.9	(1.7)	38.8	(1.1)	38.9	(1.7)	39.0	(1.2)
Birth weight; kg (N = 514)^a^	3.2	(0.5)	3.2	(0.5)	3.2	(0.5)	3.2	(0.5)	3.3	(0.4)
Maternal age (N = 501)										
<30 years	20	(9.4)	193	(90.6)	16	(7.5)	191	(89.7)	4	(1.9)
≥30 years	31	(10.8)	257	(89.2)	25	(8.7)	257	(89.2)	5	(1.7)
Paternal age (N = 495)										
<30 years	4	(4.7)	81	(95.3)	7	(8.2)	80	(94.1)	1	(1.2)
≥30 years	47	(11.5)	363	(88.5)	34	(8.3)	362	(88.3)	8	(2.0)
Small-for-gestational age infants (N = 512)										
No	46	(10.2)	404	(89.8)	38	(8.4)	402	(89.3)	9	(2.0)
Yes	6	(9.7)	56	(90.3)	5	(8.1)	56	(90.3)	0	(0)
Mode of delivery (N = 497)										
Natural labour	21	(7.9)	245	(92.1)	26	(9.8)	244	(91.7)	7	(2.6)
Assisted natural labour	15	(14.4)	89	(85.6)	6	(5.8)	88	(84.6)	2	(1.9)
Caesarean birth	14	(11.0)	113	(89.0)	9	(7.1)	113	(89.0)	0	(0)
Breast feeding (N = 507)										
No	28	(10.0)	252	(90.0)	19	(6.8)	250	(89.3)	5	(1.8)
Yes	24	(10.6)	203	(89.4)	24	(10.6)	203	(89.4)	4	(1.8)
Maternal education (N = 505)										
Grade 9 or below	22	(10.6)	185	(89.4)	16	(7.7)	183	(88.4)	3	(1.4)
Grade 10–11	24	(10.6)	202	(89.4)	21	(9.3)	202	(89.4)	4	(1.8)
Grade 12 or above	5	(6.9)	67	(93.1)	6	(8.3)	67	(93.1)	2	(2.8)
Paternal education (N = 505)										
Grade 9 or below	19	(8.5)	204	(91.5)	17	(7.6)	203	(91.0)	5	(2.2)
Grade 10–11	24	(13.0)	161	(87.0)	15	(8.1)	160	(86.5)	2	(1.1)
Grade 12 or above	8	(8.2)	89	(91.8)	11	(11.3)	89	(91.8)	2	(2.1)
Mother’s place of birth (N = 475)										
Hong Kong	30	(9.7)	279	(90.3)	28	(9.1)	278	(90.0)	5	(1.6)
Others	14	(8.4)	152	(91.6)	14	(8.4)	152	(91.6)	3	(1.8)
Father’s place of birth (N = 483)										
Hong Kong	37	(10.4)	320	(89.6)	31	(8.7)	319	(89.4)	6	(1.7)
Others	8	(6.3)	118	(93.7)	11	(8.7)	118	(93.7)	2	(1.6)
Type of birth hospital (N = 497)										
Public	38	(10.1)	340	(89.9)	31	(8.2)	338	(89.4)	7	(1.9)
Private	14	(11.8)	105	(88.2)	11	(9.2)	105	(88.2)	1	(0.8)
Household monthly income (N = 493)										
Less than HK$10,000	9	(7.7)	108	(92.3)	10	(8.5)	107	(91.5)	1	(0.9)
HK$10,000–HK$40,000	31	(10.5)	264	(89.5)	26	(8.8)	263	(89.2)	6	(2.0)
Over HK$40,000	11	(13.6)	70	(86.4)	7	(8.6)	70	(86.4)	2	(2.5)
Parents smoking pattern during the childaged 0–3years (N = 375)										
father	11	(10.3)	96	(89.7)	11	(10.3)	96	(89.7)	2	(1.9)
mother	1	(16.7)	5	(83.3)	1	(16.7)	5	(83.3)	0	(0)
both	1	(7.7)	12	(92.3)	1	(7.7)	11	(84.6)	1	(7.7)
none	22	(8.8)	227	(91.2)	17	(6.8)	226	(90.8)	5	(2.0)
Mother’s severe disease during pregnancy (N = 372)										
No	34	(9.4)	327	(90.6)	29	(8.0)	326	(90.3)	8	(2.2)
Yes	1	(9.1)	10	(90.9)	0	(0)	10	(90.9)	4	(0)
Mother’s medication during pregnancy (N = 195)										
No	21	(12.8)	143	(87.2)	13	(7.9)	143	(87.2)	4	(2.4)
Amoxicillin and/or other antibiotics	0	(0)	4	(100.0)	1	(25.0)	4	(100.0)	0	(0)
Others	2	(7.4)	25	(92.6)	1	(3.7)	25	(92.6)	1	(3.7)
Infant special medical maintenance (N = 373)										
No	26	(8.1)	294	(91.9)	25	(7.8)	293	(91.6)	7	(2.2)
Yes	8	(15.1)	45	(84.9)	5	(9.4)	45	(84.9)	1	(1.9)
Child severe disease 0–3 years old (N = 508)										
No	50	(11.2)	395	(88.8)*	42	(9.4)	394	(88.5)*	7	(1.6)
Yes	1	(1.6)	62	(98.4)*	2	(3.2)	61	(96.8)*	2	(3.2)
Medication 0–3 years old (N = 133)										
No	10	(15.2)	56	(84.8)	3	(4.5)	56	(84.8)	1	(1.5)
Amoxicillin and/or other antibiotics	3	(7.5)	37	(92.5)	5	(12.5)	37	(92.5)	1	(2.5)
Others	3	(11.1)	24	(88.9)	1	(3.7)	24	(88.9)	1	(3.7)
Dental trauma 0–3 years old (N = 373)										
No	33	(8.9)	337	(91.1)	30	(8.1)	336	(90.8)	8	(2.2)
Yes	1	(33.3)	2	(66.7)	0	(0)	2	(66.7)	0	(0)
Tooth extraction due to caries 1–3 years old (N = 373)										
No	33	(8.9)	328	(91.1)	29	(8.1)	327	(90.8)	8	(2.2)
Yes	1	(33.1)	11	(84.6)	1	(7.7)	11	(84.6)	0	(0)
Age begin to use toothpaste (N = 507)										
Before 4 years old	41	(10.2)	359	(89.8)	35	(8.8)	357	(89.2)	7	(1.8)
4 years old or older	10	(9.3)	97	(90.7)	8	(7.5)	97	(90.7)	2	(1.9)
Toothpaste type before 5 years old (N = 506)										
Adult toothpaste	3	(7.7)	36	(92.3)	2	(5.1)	36	(92.3)	0	(0)
Children toothpaste	48	(10.3)	419	(89.7)	41	(8.8)	417	(89.3)	9	(1.9)
Toothpaste contained fluoride before5 years old (N = 506)										
No	6	(12.0)	44	(88.0)	3	(6.0)	44	(88.0)	0	(0)
Yes	16	(10.6)	135	(89.4)	10	(6.6)	135	(89.4)	5	(3.3)
Don’t know	29	(9.5)	276	(90.5)	30	(9.8)	274	(89.8)	4	(1.3)
Amount of tooth paste applied before 5 years old(N = 500)										
Smear	13	(8.7)	136	(91.3)	16	(10.7)	136	(91.3)	1	(0.7)
Pea size	32	(10.6)	271	(89.4)	23	(7.6)	269	(88.8)	5	(1.7)
Larger than pea size	6	(12.5)	42	(87.5)	3	(6.2)	42	(87.5)	3	(6.2)
Toothbrushing habits before 5 years old(N = 499)										
Less than once daily	3	(7.9)	35	(92.1)	2	(5.3)	35	(92.1)	1	(2.6)
At least once daily	48	(10.4)	413	(89.6)	39	(8.5)	411	(89.2)	8	(1.7)

*p<0.05.

mean (SD)^a^ or frequency distribution, n (%).

In the unadjusted logistic regression model (Table 3), only the independent variable of ‘child severe disease 0–3 years old’ was associated with the occurrence of ‘any defect’. Subjects with experience of severe diseases during the 0–3 years period were 7.89 times more likely to be affected by ‘any defect’ compared to those children who did not have the experience (OR 7.89; 95% CI 1.07, 58.14; p = 0.043). No associations were found between any of the three main types of DDE and various independent variables in the unadjusted model. After controlling for potential confounding factors in the adjusted model, ‘child severe disease 0–3 years old’ was no longer associated with the presence of ‘any defect’. As a result, no variable remained significant in the final adjusted model. Poisson regression models confirmed the above results for the main types of demarcated opacities and hypoplasia.

**Table 3:** Multinomial logistic regression of DDE.

	Unadjusted^1^			Adjusted^2^		
Variable	OR	95% C.I.	p-value	OR	95% C.I.	p-value
Any defects						
Child severe disease 0–3 years old						
no	1			-	-	-
yes	7.89	1.07, 58.14	0.043*	-	-	-

1 Unadjusted: separate logistic regression analysis.

2 Adjusted: adjusted for gender; gestation age; birth weight; maternal age (<30 years, ≥30 years); paternal age (<30 years, ≥30 years); small-for-gestational age infants (no, yes); mode of delivery (natural labour, assisted natural labour, caesarean birth); breast feeding (no, yes); maternal education (grade 9 or below, grade 10–11, grade 12 or above); paternal education (grade 9 or below, grade 10–11, grade 12 or above); mother’s place of birth (Hong Kong, outside Hong Kong); father’s place of birth (Hong Kong, outside Hong Kong); type of birth hospital (public, private); household monthly income (less than HK$10,000, HK$10,000–HK$40,000, over HK$40,000); parent smoking pattern during the child aged 0–3 years (father, mother, both, and none); mother’s severe disease during pregnancy (no, yes); mother’s medication during pregnancy (no, amoxicillin and/or other antibiotics, and others); infant special medical maintenance (no, yes); child severe disease 0–3 years old (no, yes); medication 0–3 years old (no, amoxicillin and/or other antibiotics, and others); dental trauma 0–3 years old (no, yes); tooth extraction due to caries 1–3 years old (no, yes); age begin to use toothpaste (before 4 years old, 4 years old or older); toothpaste type before 5 years old (adult toothpaste, children toothpaste); toothpaste contained fluoride before 5 years old (no, yes); amount of toothpaste applied before 5 years old (smear, pea size, larger than pea size); tooth brushing habits before 5 years old (less than once daily, at least once daily).

*p<0.05.

## Discussion

The present prospective cohort study collected the subjects’ background information and health care related records from a random community sample of Hong Kong children born in 1997, and the prevalence of DDE was assessed when the subjects were aged 12-year-old. Bivariate analyses were performed to screen for possible aetiological factors responsible for the presence of DDE and subsequent multinomial logistic regression was undertaken to control for potential confounding factors.

To date, most studies that investigated risk factors of DDE were case-control studies [10], [11], [16] or cross-sectional surveys [14], [17], and the subjects’ information was collected retrospectively. Recall bias was thereby introduced into these studies. In a carefully designed study by Arrow [17], information was collected prior to eruption of the teeth of interest. While reporting bias was avoided, recall bias still existed due to the retrospective nature of the study. To the best of the authors’ knowledge, there is only one antecedent prospective study that investigated the potential causal factors for DDE. A group of 696 New Zealand children participated in the Dunedin Multidisciplinary Child Developmental Study in which the medical and dental histories were correlated with the presence of DDE; only chicken pox before the age of 3 years and trauma to the primary incisors were identified as risk factors [12]. However, only bivariate analyses were undertaken without control of other potential confounding factors. Moreover, there might have been dramatic changes over the past two decades in the pattern of children’s health profiles due to immunization, the use of medication and readily available medical facilities. Therefore, it is difficult to compare the present results with those of other older studies.

Male subjects were found at an increased risk of demarcated opacities (Table 2). We speculate that this might have arisen because of the small number of subjects with demarcated opacities in our sample. Results on gender differences of DDE have been inconsistent and warrant further exploration. While some researchers found male subjects more likely to be affected [22], others [14], [17] reported no gender differences.

The associations of DDE with birth characteristics, including gestational weeks, birth weight, mode of delivery and parental age, were not significant (Table 2). Our results confirmed findings from previous studies [16], [23], but were in contrast with a pilot study by van Amerongen and Kreulen [24], where subjects born prematurely were common among the subjects affected by enamel defects. Since no premature babies were recruited in the current study, further research is required to clarify the association of DDE with gestational weeks.

Investigations into the effect of breastfeeding on DDE are interesting. Developing enamel has been reported to be especially sensitive to even a minute amount of dioxin [25], which is expressed in breast milk [26]. Duration of breastfeeding has been associated with the presence of DDE in permanent teeth [26] and DDE have even been suggested as a biomarker of dioxin exposure [27]. However, our results (Table 2), which are in agreement with several other epidemiologic studies [14], [16], [17], suggested that the duration of breastfeeding did not affect the occurrence of DDE.

Since our subjects were drawn from a random community sample, we expect the socio-economic status, including the levels of parental education and household monthly incomes, of our subjects to be proportionately distributed and so be a reflection of the society’s socio-economic status as a whole. Even though there were reports stating that subjects of lower socio-economic status had a higher chance of developing DDE [10], [28], we were unable to find significant associations in this study. Thus our results were in line with those reported by Arrow [17] and other researchers [29], [30].

Contrary to the findings reported by Ford and colleagues [10], which indicated that parental cigarette smoking was correlated with DDE, we failed to identify any statistically significant associations between DDE and any of the prenatal exposure factors, such as parental smoking, maternal severe disease and medication during pregnancy. Early life health problems have consistently been reported to be associated with DDE. Arrow [17] found subjects who had had an illness during the neonatal period were at increased risk of DDE. Health problems in the first three years of life were also found to increase the likelihood of DDE [11], [14]. Data on severe diseases experienced by children during the first three years of life were collected in this study because this is the period when the index teeth were undergoing formation [31], [32] overlaps with the time period when children are most susceptible to various diseases. As a result, we revealed the significant effects of ‘child severe disease 0–3 years old’ on ‘any defect’ and diffuse opacities. However, this association disappeared in the adjusted logistic model when confounding factors were controlled for.

Fluoride consumption has been related to the occurrence of DDE in terms of diffuse opacities [2], [33] and a significant association between the use of adult toothpaste and DDE was reported from a case-control study that was conducted in Australia [10]. Therefore, oral hygiene habits such as the age when toothpaste usage commenced, the type of toothpaste, amount of toothpaste applied, and the frequency of tooth brushing were studied in this investigation. We found no significant associations between the various oral hygiene habits and the occurrence of DDE. Although the children in this study who used an adult type toothpaste before 5 years of age were more susceptible to ‘any defect’ and diffuse opacities than those who used a child formula toothpaste, the result was not statistically significant (p>0.05).

Inevitably the present study had several limitations. Due to the low prevalence of demarcated opacities and hypoplasia in the Hong Kong population, the number of subjects affected by these two types of DDE was very limited. This decreased the statistical power of the analyses; therefore, the findings on demarcated opacities and hypoplasia should be interpreted with caution. Future research into the aetiological factors of demarcated opacities and hypoplasia in situations where the prevalence is rather low could adopt a case-control study as the study design. The neonatal period is a very important stage of a child’s development. Under the item ‘child severe disease 0–3 years old’, we did not differentiate between the neonatal period and the remainder of this age range. In addition, while different types of diseases experienced by the children were recorded, statistical analysis was not performed with respect to each specific disease type. This may explain why the impact of childhood severe disease on DDE disappeared after controlling for potential confounding factors. Future studies could be designed to gather specific detailed information and other potential risk factors for DDE. Furthermore, investigations into the effect of antibiotics on DDE are not straightforward because the illness for which the antibiotic was prescribed may have caused the DDE and thereby serve as a confounding factor.

## Conclusions

Our uniquely large prospective cohort study among randomly selected Hong Kong children expands the current knowledge on the aetiology of DDE. Experiences of severe diseases experienced during 0–3 years of age were associated with ‘any defect’ and diffuse opacities. Children with severe diseases in early life were found to be almost 8 times more likely than healthy children to be affected by DDE. However, after adjusting for confounding factors, no variable was associated with the occurrence of DDE.
